# Application of Hydrogen Peroxide as an Innovative Method of Treatment for *Legionella* Control in a Hospital Water Network

**DOI:** 10.3390/pathogens6020015

**Published:** 2017-04-17

**Authors:** Beatrice Casini, Francesco Aquino, Michele Totaro, Mario Miccoli, Irio Galli, Laura Manfredini, Carlo Giustarini, Anna Laura Costa, Benedetta Tuvo, Paola Valentini, Gaetano Privitera, Angelo Baggiani

**Affiliations:** 1 Department Translational Research, N.T.M.S., University of Pisa, via S. Zeno 35-37, 56127 Pisa, Italy; cheps86@hotmail.it (F.A.); micheleto@hotmail.it (M.T.); alauracosta@alice.it (A.L.C.); tuvobenedetta@hotmail.it (B.T.); paola.valentini@dps.unipi.it (P.V.); gaetano.privitera@med.unipi.it (G.P.); angelo.baggiani@med.unipi.it (A.B.); 2 Department of Clinical and Experimental Medicine, University of Pisa, 56127 Pisa, Italy; mario.miccoli@med.unipi.it; 3 Azienda USL6 Livorno, via Forlanini 26, 57125 Piombino, Livorno, Italy; i.galli@usl6.toscana.it (I.G.); l.manfredini@usl6.toscana.it (L.M.); carlo.giustarini@legalmail.it (C.G.)

**Keywords:** hydrogen peroxide, *Legionella*, hospital, disinfection

## Abstract

Objectives: To evaluate the effectiveness of hydrogen peroxide (HP) use as a disinfectant in the hospital water network for the control of *Legionella* spp. colonization. Methods: Following the detection of high levels of *Legionella* contamination in a 136-bed general hospital water network, an HP treatment of the hot water supply (25 mg/L) was adopted. During a period of 34 months, the effectiveness of HP on *Legionella* colonization was assessed. *Legionella* was isolated in accordance with ISO-11731 and identification was carried out by sequencing of the *mip* gene. Results: Before HP treatment, *L. pneumophila* sg 2–15 was isolated in all sites with a mean count of 9950 ± 8279 cfu/L. After one-month of HP treatment, we observed the disappearance of *L. pneumophila* 2–15, however other *Legionella* species previously not seen were found; *Legionella* *pneumophila* 1 was isolated in one out of four sampling sites (2000 cfu/L) and other non-*pneumophila* species were present in all sites (mean load 3000 ± 2887 cfu/L). Starting from September 2013, HP treatment was modified by adding food-grade polyphosphates, and in the following months, we observed a progressive reduction of the mean load of all species (*p* < 0.05), resulting in substantial disappearance of *Legionella* colonization. Conclusion: Hydrogen peroxide demonstrated good efficacy in controlling *Legionella*. Although in the initial phases of treatment it appeared unable to eliminate all *Legionella* species, by maintaining HP levels at 25 mg/L and adding food-grade polyphosphates, a progressive and complete control of colonization was obtained.

## 1. Introduction

*Legionella* spp. is a Gram-negative aerobic opportunistic bacterium, responsible for a severe form of pneumonia called Legionnaires’ disease (LD) [1]. It is a waterborne human pathogen frequently associated with nosocomial infections, particularly among immunosuppressed patients such as those with acquired immune-deficiency syndrome (AIDS), transplant patients, and those undergoing aggressive chemotherapy [2].

The case fatality rate of LD associated with outbreaks is lower than that of sporadic cases, generally around 8–15%, but it can be higher, especially in the case of hospital-acquired infections, AIDS patients, transplant patients, and those undergoing aggressive chemotherapy [3]. Furthermore, recent studies report an underestimated *Legionella* risk in large and ancient water networks of community settings as hotels, tourist facilities and residential buildings [4].

The genus *Legionella* includes 57 species (including subspecies) and about 70 serogroups, not all associated with human disease. The species most frequently detected in diagnosed cases [5] is *Legionella pneumophila*, consisting of 16 serogroups; *Legionella pheumophila* serogroup 1, responsible for the first identified outbreak, occurred in 1976 in Philadelphia (USA), is the cause of 95% of infections in Europe and 85% in the world [6].

In Italy, 1497 cases were reported in 2014 and only 62 (4.1%) of them were hospital-acquired infections, although among the latter, the highest case fatality rate was registered, 30.8% compared to 10.1% of community infections. The species of *Legionella* implicated in all cases was *Legionella pneumophila* [7].

*Legionella* colonization control in hospital hot water networks is an important patient safety-related target but, at the same time, it represents a challenge for finding a cost-effective solution.

Several concerns are related to *Legionella* risk control; the complexity of the water system structure in huge buildings with the possibility, in some branches, of low water flow and a higher likelihood of biofilm development. Old pipeline systems frequently show high corrosion levels that provide a favorable substrate for bacterial growth, and this presents the need for operating with sanitation interventions. Hot water systems, which usually operate at 40–50 °C, provide the ideal growth conditions for *Legionella*, whose optimal temperature for in vitro growth is 36 °C (and a range of 15–43 °C), with a generation time of 99 min under optimal conditions [8,9,10].

The second important issue is represented by intrinsic bacterial features. *Legionella* is a resistant microorganism, able to survive in a wide range of natural and artificial environments [11], including adverse environmental conditions in quiescence, and can subsequently recover activity and pathogenicity. Similarly, *Legionella* demonstrates resistance to chemical agents at concentrations usually applied for water disinfection.

The first comprehensive review of disinfection methodologies was published in 1990 [12] and the most recent in 2011 [13]; no evidence-based recommendation can yet be made for any of the potentially applicable treatments to the hospital water network so that the Centers for Disease Control and Prevention Guidelines for Preventing Health-Care-Associated Pneumonia suggests the validation of decontamination procedures through collection of specimens for culture at two-week intervals for three months after treatment to ensure the effectiveness of the institution’s safety practices [14,15,16].

Several chemical products, mostly chlorine-based, have been employed as water disinfection systems; all of them are useful for the control of *Legionella* mean count, but eradication of colonization is incomplete. Bacteria can shelter in biofilms, and free living protozoa, especially cysts, can protect bacteria against disinfectants [17,18,19,20,21]. In particular, amoebae play a role in re-emergence of *L. pneumophila* in water sources after disinfection, since the bacteria can be protected by the trophozoites and/or the cysts forms of amoebae [22].

As demonstrated by several studies, complete eradication of *Legionella* from hospital water network systems seems impossible to achieve, even with long-term disinfection [23,24,25], and in high risk areas, it is necessary to install point-of-use filters to prevent hospital infections [26].

This highlights the need for the evaluation of new disinfectant products to obtain better results in disinfection efficacy and cost-effectiveness [24].

Recently, HP was applied for hot water disinfection in some hospitals. Silver-stabilized HP has a history of use in the control of *Legionella* in water systems, but only a few experimental studies have tested HP alone (in the water system, and in cleaning baths for dental and medical instruments); this topic is now regaining a role of central interest.

Literature data report several references about the properties, the germicidal effectiveness, and the potential uses for HP in healthcare water networks. Published reports ascribe HP as a good reagent for bactericidal, virucidal, and fungicidal activity.

Continuous treatment of water distribution systems using HP, generally in combination with silver salts, is now allowed in many countries. In France, the Ministère de la Santé Publique considers this formulation as part of the guidelines for the treatment of *Legionella pneumophila* [27].

HP is a strong oxidizing agent that oxidizes microorganisms’ enzymatic systems, releasing free oxygen atoms (nascent oxygen). HP production occurs naturally in the human body as a defense against antigens.

HP as a water disinfectant offers various advantages. It is a strong oxidizer, bactericidal at 3% solution (D value *E. coli*: 0.57 min), a sterilant at 6% with six hours of exposure, more powerful than chlorine dioxide, and more stable at high temperatures and pH compared to chlorine-based disinfectants.

Furthermore, HP is non-toxic to humans and the environment, tasteless, and is not mutagenic or carcinogenic.

HP has the ability to form powerful oxidants such as hydroxyl radical and singlet oxygen. They appear to present no danger by toxicity or other hazards, when diluted in water to their effective concentration as a disinfectant. HP decomposes rapidly in different environmental compartments, due to biotic degradation (microbial catalase and peroxidase enzymes), and abiotic degradation (transition metal, heavy metal, oxidation or reduction reactions, with organic compounds or inorganic substances). The lack of toxicity of HP to people and animals and its lack of environmental impact has been confirmed by the U.S. Food and Drug Administration (FDA) and the U.S. Environmental Protection Agency [28]. European directives do not establish a concentration limit for HP in drinking water, although the German and British version of the EN 902: 2016: “Chemicals used for treatment of water intended for human consumption. Hydrogen peroxide” provides a dosage up to 17 mg/L.

HP still presents numerous advantages from both an economic and operative point of view; operating costs are much lower than traditional water disinfection systems, as are investment costs and expenditure for equipment. HP solution is readily available and can be stored long-term (maximum loss of concentration 3% per year). Moreover, it has a low corrosive effect, while pipeline corrosion is a frequent and no-negligible problem with chlorine-based disinfection systems. To prevent corrosion polyphosphate compounds may be added during disinfection, although they can remove metals. Removal of metals would lead to *Legionella* deficient in superoxide dismutase activity and thereby increased susceptibility to toxic oxygen metabolites, including hydrogen peroxide. Superoxide dismutases (SODs) are metalloenzymes that catalyze the decomposition of superoxide radical. Although SODs of the manganese (MnSOD) or iron (FeSOD) class are widespread among bacteria, SOD containing both copper and zinc (CuZnSOD) has been demonstrated in a small number of gram-negative bacterial species, such as *Legionella pneumophila*. CuZnSOD plays a role in the survival of *L. pneumophila* during the stationary phase of growth [29].

HP-based disinfection has recently been applied, with satisfactory results in the reduction of microbial contamination in dental unit water; recent studies have demonstrated the efficacy of HP treatment versus several microorganisms, including *Legionella* [30,31,32].

## 2. Results

Before the start of the HP treatment, high *Legionella* concentrations were detected in all the examined water points. Concentrations ranged from 3000 to 20,800 cfu/L, with a mean value of 9950 ± 8279 cfu/L in flushed water samples, indicating significant colonization of the entire hospital building water network (sampled 3 July 2013). The bacterial species was identified as *Legionella pneumophila* 2–15.

This potentially critical situation led to the application of a HP-based disinfection strategy on 19 July 2013, commencing with a “shock treatment” of the water network using a HP solution of 100 mg/L and silver ions at 100 µg/L for 12 h, followed by a continuous treatment with 10 mg/L of HP and 10 µg/L of silver ions.

On 29 July 2013, an initial sampling was performed after the beginning of the treatment. Culture analysis demonstrated the absence of *Legionella pneumophila* 2–15 in all four sampling sites. However, *Legionella* species not previously cultured before was present: *Legionella pneumophila* 1 was isolated in one out of four sampling sites (2000 cfu/L) and other non-*pneumophila* species were present in all the sites (mean load 3000 ± 2887 cfu/L).

Samples tested by PCR showed results positive for the presence of *Legionella*, and where non-*pneumophila Legionella* species were identified, the sequence of the *mip* gene showed the presence of *Legionella longbeachae* serogroup 1 (strain NSW150).

These results were considered unsatisfactory, and led to a change in disinfectant product; from September 2013, HP and silver ions were replaced by a formulation of HP only, at higher concentration (25 mg/L), and with the addition of polyphosphates as a film-forming product.

This disinfection strategy showed tangible results after six months of treatment.

The efficacy of the new disinfection was observed from January 2014; a progressive reduction of non-*pneumophila Legionella* species loads to less than 500 cfu/L occurred, and the complete disappearance of *L. pneumophila* 1 was observed.

In July 2014, a reduction in HP concentration from 25 to 10 mg/L, probably due to a failure in the HP dispensing device, caused an increase in *Legionella* concentrations and the reappearing of over 3000 cfu/L *L. pneumophila* 2–15. However, the mean bacteria load remained lower compared to the initial situation (800 ± 1524 cfu/L for *L. pneumophila* 2–15 and 220 ± 178 cfu/L for non-*pneumophila Legionella*).

With the HP concentration maintained at 25 mg/L, at the subsequent sampling test (November 2014), all collected samples scored negative for *Legionella*, except for the site on the fourth floor in Obstetrics and Gynecology.

**Figure 1:**
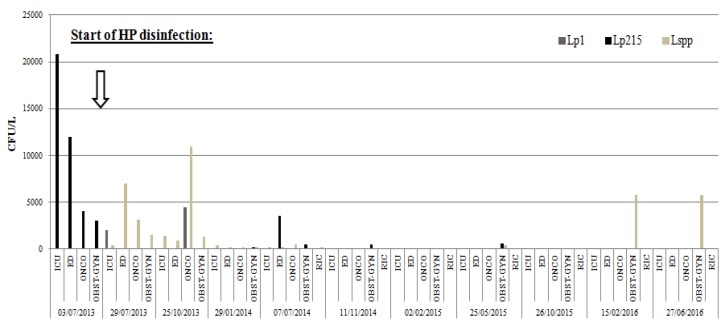
*Legionella pneumophila* sg1 (Lp1), *Legionella pneumophila* sg2-15 (Lp2-15), *Legionella* spp. (*L* spp.) loads (cfu/L) detected in the four different sampling sites in the hospital. Intensive Care Unit (ICU), Emergency Department (ED), Oncology (ONCO), Obstetrics and Gynecology (OBST-GYN), Recirculation point (RIC) during the period of hydrogen peroxide (HP) disinfection (from July 2013 to June 2016).

**Figure 2:**
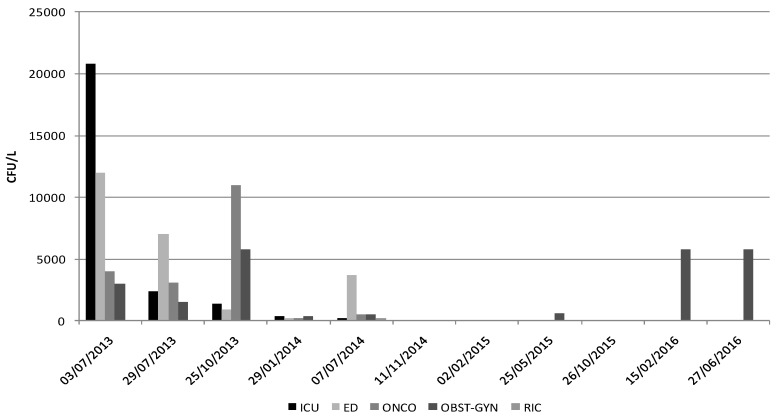
*Legionella* loads (cfu/L) detected in each of the four sampling sites in the hospital: Intensive Care Unit (ICU), Emergency Department (ED), Oncology (ONCO), Obstetrics and Gynecology (OBST-GYN), Recirculation point (RIC) during the period from July 2013 to June 2016.

In the following months, five different sampling tests were performed; February 2015, May 2015, October 2015, February 2016 and June 2016. The sampling tests showed the absence of *Legionella* contamination in all sites, with the exclusion of two instances, in May 2015 (600 cfu/L of *L. pneumophila* 2–15) and June 2016 (400 and 5800 cfu/L of species non-*pneumophila*.) when *Legionella* was again detected in the washbasin on the fourth floor (Figure 1 and Figure 2). The higher levels of contamination in this point may be due to its location, as the most distal area in the water network from the HP dispensing device, and due to its infrequent use and consequent water stagnation in the terminal portion.

Statistical analysis demonstrated that the abatement in *Legionella* loads after HP treatment was significant. Application of the Nemenyi test gave significant p-values from a comparison of the latest test to sampling on November 2014 (*p*-value < 0.05) (Table 1).

The application of the new disinfectant formulation with 25 mg/L HP and the addition of filming product demonstrated a positive effect on corrosion reduction, suggesting that decreased intervention for water system maintenance is possible with correct treatments. The detection of dissolved iron concentrations was used as a proxy for pipelines corrosion; this was influenced by seasonal variability, due to different sources of water supplied from the municipal aqueduct (an additional water supply is required in summer). In autumn, sampling displayed the highest iron ion values compared to other periods of the year, despite a trend in reduction of iron ion values during the monitoring period, suggesting a possible decrease of corrosion levels. The same trend was observed with turbidity (Table 2). Further physico-chemical parameters (temperature, pH, conductivity) and detected HP concentration as assessed at point of use, are shown in Table 3.

## 3. Discussion

This is one of the few studies of its kind performed in a hospital setting, although previous experiences reported in the literature used HP either as a formulation with silver salts, or with acetic and peracetic acid, in different concentrations.

In an Israeli study, performed on a 50-bed ward of a great hospital, a formulation of HP and silver salts applied for 24 months, demonstrated a significant reduction in *Legionella* contamination, from mean count values of 200–14,000 cfu/L to total absence [33].

Another two experimentations, performed in Italian long-term care facilities, did not yield a total abatement of the contamination, although an approximate 2-log reduction in *Legionella* mean count was attained [34,35].

Remarkable results were obtained in a recent study by Modena-Reggio Emilia University (Italy), from using a treatment based on the association of HP with acetic and peracetic acid, performed on a highly-contaminated building. Initially, *Legionella* counts were rapidly reduced, remaining stable at low levels in the first seven months, until complete removal of bacteria in more than 90% of samples during the subsequent eighth month. During the treatment, an interesting observation was the progressive substitution of *L. pneumophila* (serogroups 1, 6, 9) with environmental species of *Legionella*, such as *L. jamestowniensis* [36].

The results of this 36-month field study performed at the Villafranca General Hospital demonstrated good efficacy of HP treatment in controlling *Legionella*. In particular, the dosage of HP 25 mg/L with the addition of polyphosphates emerged as a valid alternative to the more frequent use of silver salts.

Overall, our data suggest that the effectiveness of HP is evident in the long-term. In the early stages of treatment, some *Legionella* species other than *L. pneumophila* appear to resist treatment by disinfectant and subsequently replace *L. pneumophila*; these species are less pathogenic and are almost never associated with human disease. *Legionella longbeachae* was identified as responsible for 43 cases between 2005 and 2012 by the European Surveillance System (Tessy) and for a cluster of six cases from an amateur gardener in 2013 in Scotland [37], although infection by this species is unusual. These findings demonstrate the importance of accurate environmental surveillance in order to monitor the presence of all *Legionella* species, including non-pneumophila ones.

This characteristic trend, with a first stage of *L. pneumophila* reduction followed by its substitution by less pathogenic *Legionella*, and long-term efficacy in the reduction of all *Legionella* species, was highlighted by Marchesi et al. [36], in the use of HP disinfection alongside peracetic acid.

As with other water disinfection methods, it was demonstrated that maintaining an appropriate and uninterrupted concentration of HP was very important, as new increases of *Legionella* loads were observed during the study period due to a lower doses of disinfectant. Through our data, we can assert that 25 mg/L HP levels ensure good control of *Legionella* colonization and its almost complete disappearance in the long-term, qualifying this method as a reliable new option for water treatment.

This study is the first to evaluate the application of HP as the only disinfectant in *Legionella* colonization control, and demonstrated good performance using HP-based disinfection. This product could represent a valid alternative to chlorine-based disinfectants, with at least comparable results in efficacy, and advantages in cost reduction, with direct costs for installation of the dosing device and disinfectant, and indirect costs associated with the reduction of pipeline corrosion and maintenance fees.

## 4. Materials and Methods

### 4.1. Setting

The Villamarina General Hospital of Piombino (Leghorn, Italy), is a 136-beds hospital (120 ordinary and 16 day hospital) with a catchment area of about 60,000 inhabitants. Built in the early 1990s as an extension of a previous structure, it has been active since 1992.

The architectural structure of the hospital is a monoblock with a central plate with three levels (−2, −1, ground floor) and three vertical towers, one containing five floors and the other two containing four floors.

The reference specialties are: Cardiology, General Medicine, Oncology, General Surgery, Urology, Orthopedics, Ophthalmology, Obstetrics and Gynecology, Pediatrics.

In July 2013, within the water safety plan (WSP) implementation program, hot water system disinfection, and a systematic monitoring program with sampling of final points of use within the water network was commenced.

### 4.2. Water Disinfection

Considering the water network characteristics, with old and galvanized steel-made pipelines, the need to minimize the risk of corrosion and related maintenance costs led to the discarding of chlorine dioxide disinfection methods and the uptake of HP as a disinfection product.

The first performed action was a “shock” disinfection with a solution of HP and silver ions for 12 h. Subsequently, a continuous disinfection system, based on a formulation of 10 mg/L HP and 10 µg/L silver ions, at points of use, was applied.

In September 2013, the HP-silver ions disinfection was replaced by the use of HP alone with 25 mg/L at points of use. Furthermore, polyphosphates were added to disinfectant as a film-forming product to reduce pipeline corrosion. This formulation was employed from September 2013 to June 2016, with the only exception of July 2014, when HP concentration went down to 10 mg/L, probably due to a dosing device malfunction.

### 4.3. Sample Collection and Detection of Legionella *spp.*

Between July 2013 and June 2016, 59 hot water samples were collected. Four different target points, one for each floor, were selected, considering their degree of representation of the hospital water network: an emergency room bathroom (basement), an intensive care room (ground floor), an Oncology Day Hospital room (first floor) and the hand basin of the Obstetrics and Gynecology delivery room (fourth floor). Since July 2014, a fifth sampling point was added at the circulation circuit of the hot water network.

Water samples were assayed for *Legionella* and tested for the following physico-chemical parameters: temperature (°C), pH (pH units), conductivity (µS/cm), turbidity (NTU), iron ions (µg/L Fe). Turbidity and iron ions were assayed only for two sampling points, at the intensive care room (ground floor), and at the Oncology Day Hospital room (first floor).

At the same time, the disinfectant concentration (hydrogen peroxide, or hydrogen peroxide and silver ions during the period of application) in water was regularly measured at the point of use to determine an accurate indication of HP levels being introduced into the water supply. The HP concentration was determined by colorimetric test Merckoquant^®^ test strips.

During the first sampling test, to detect basal levels of *Legionella* colonization, two water samples were collected from each sampling point, one immediately and one after 5 min of flushing. In the subsequent sampling tests, only flushed samples, that were more representative of overall water network contamination levels, were collected.

The isolation of *Legionella* spp. in hot water samples was performed in accordance with standard procedures [38,39]. One liter of water was filtered through a membrane with a porosity of 0.2 µm diameter (Millipore, Billerica, MA, USA). After filtration, the membrane was immersed in 10 mL of the same water and subjected to sonication for 5 min, allowing the detachment of cells from the membrane and their suspension in water. The suspension was subjected to a thermal inactivation treatment at 50 °C for 30 min with the aim to select for *Legionella* spp., while inactivating all microbial species not resistant to high temperature. Afterwards, 0.1 mL of the suspension was seeded on *Legionella* BMPA selective medium (Oxoid Ltd., Basingstoke, Hampshire, UK), and the plates were incubated at 37 °C for 7–10 days within jars under a modified atmosphere (2.5% CO_2_) Finally, *Legionella* colonies grown on the medium were subjected to species and serogroup identification analysis using a multi-purpose latex agglutination test (*Legionella* Latex Test, Oxoid Ltd., Basingstoke, Hampshire, UK). Identification of *Legionella* species was carried out by sequencing of the *mip* gene (558 bp) [40].

DNA template was prepared by resuspending the colony in 500 µL of sterile water and incubating at 99 °C for 10 min. For each, PCR using 50 µL of mix were prepared with 31.25 µL of water; 5 µL of 10X PCR Buffer (15 mM MgCl2), 1 µL of dNTP mix (10 µM); 1.25 µL of mip595R 5′-CATATGCAAGACCTGAGGGAAC (20 mM); 1.25 µL of mip58F 5′-GCTGCAACCGATGCCAC (20 mM); 0.25 µL of HotStarTaq DNA Polymerase (5 U/µL); and 10 µL of extract (HotStarTaq DNA Polymerase, Qiagen, Germantown Road, MD 20864 USA). PCR reaction steps were as follows: initial denaturation at 95 °C for 15 min; denaturation at 94 °C for 1 min; annealing at 55 °C for 1 min; extension at 72 °C for 1 min; termination at 72 °C for 10 min. Denaturation to extension steps were repeated 35 times. 10 µL of the amplified PCR mixture was loaded to a 1% agarose gel with ethidium bromide. A 1.5 Kb ladder was used to compare amplified PCR product.

After the electrophoretic run, (110 V for 30 min), *mip* gene amplification results were visualized in a UV transilluminator.

Amplified *mip* gene was sequenced by outsourcing (GATC, Biotech, Jakob-stadler-plaz 778467 Constance, Germany), and sequence alignment was performed by BioEdit Version 7.0.0. Sequences identification was obtained using a Basic Local Alignment Search Tool (BLAST) Database.

### 4.4. Statistical Analysis

Data are shown as means, standard deviations and medians. Normality of distribution was assessed using the Kolmogorov-Smirnov test, the variable was not Gaussian. The Nemenyi test for paired data was performed to compare the values of different times. Post-hoc power tests were conducted to estimate the sample size, 1-beta values of significant data were >0.8, assuring an appropriate sample size. The statistical analysis was carried out using the IBM SPSS software package, version 17.0.1 (IBM, Armonk, NY, USA).

## Figures and Tables

**Table 1:** Statistical comparison between *Legionella* loads detected in every date of sampling.

*p*-Values	T0 3 July 2013	T1 29 July 2013	T2 25 October 2013	T3 29 January 2014	T4 7 July 2014	T5 11 November 2014	T6 2 February 2015	T7 25 May 2015	T8 26 October 2015	T9 15 February 2016	T10 27 January 2016
**T0—3 July 2013**	1	0.670	0.790	0.136	0.241	0.003 *	0.003 *	0.012 *	0.003 *	0.003 *	0.033 *
**T1—29 July 2013**	0.670	1	0.873	0.286	0.456	0.011 *	0.011 *	0.038 *	0.011 *	0.011 *	0.088
**T2—25 October 2013**	0.790	0.873	1	0.220	0.365	0.007 *	0.007 *	0.025 *	0.007 *	0.007 *	0.062
**T3** **—29 January 2014**	0.136	0.286	0.220	1	0.749	0.136	0.136	0.311	0.136	0.136	0.522
**T4** **—7 July 2014**	0.241	0.456	0.365	0.749	1	0.070	0.070	0.183	0.070	0.070	0.337
**T5** **—11 November 2014**	**0.003**	**0.011**	**0.007**	0.136	0.070	1	1.000	0.631	1.000	1.000	0.394
**T6** **—2 February 2015**	**0.003**	**0.011**	**0.007**	0.136	0.070	1.000	1	0.631	1.000	1.000	0.394
**T7** **—25 May 2015**	**0.012**	**0.038**	**0.025**	0.311	0.183	0.631	0.631	1	0.631	0.631	0.709
**T8** **—26 October 2015**	**0.003**	**0.011**	**0.007**	0.136	0.070	1.000	1.000	0.631	1	1.000	0.394
**T9** **—15 February 2016**	**0.003**	**0.011**	**0.007**	0.136	0.070	1.000	1.000	0.631	1.000	1	0.394
**T10** **—27 June 2016**	**0.033**	0.088	0.062	0.522	0.337	0.394	0.394	0.709	0.394	0.394	0

(*) Statistical significance (*p*-value: *p* < 0.05).

**Table 2:** Iron ions (µg/L Fe) and nephelometric turbidity unit (NTU) values detected in the Intensive Care Unit (ICU) and Oncology (ONCO) during the period of the study, from July 2013 to June 2016. Not applied (n.a).

	3 July 2013	29 July 2013	25 October 2013	29 January 2014	7 July 2014	11 November 2014	2 February 2015	25 May 2015	26 October 2015	15 February 2016	27 June 2016
**ICU**											
**Iron Ions (µg/L Fe)**	140	n.a	300	140	120	210	<40	50	184	80	n.a
**Turbidity (NTU)**	2.74	1.62	2.75	3.92	4.18	2.6	<0.5	1.13	n.a	0.66	< 0.5
**ONCO**											
**Iron Ions (µg/L Fe)**	180	n.a	230	50	70	230	40	<40	144	<40	n.a
**Turbidity (NTU)**	6.73	0.85	0.73	1.34	0.36	2.68	<0.5	<0.5	0.88	0.87	1.42

**Table 3:** Values of temperature (°C), pH (pH units), conductivity (µS/cm) and hydrogen peroxide (HP) concentration (mg/L) at point of use from July 2013 to June 2016. Intensive Care Unit (ICU), Emergency Department (ED), Oncology (ONCO), Obstetrics and Gynecology (OBST-GYN), Recirculation point (RIC); not applied (n.a).

	3 July 2013	29 July 2013	25 October 2013	29 January 2014	7 July 2014	11 November 2014	2 February 2015	25 May 2015	26 October 2015	15 February 2016	27 June 2016
**ED**											
**Temperature (°C)**	48.1	42.6	37.6	42.4	42	41	40	42.5	42.7	40	46.3
**pH (pH units** **)**	7.4	7.2	7.6	7.6	7.4	7.6	7.6	7.7	7.6	7.6	7.6
**conductivity (µS/cm)**	1394	1365	761	880	865	886	945	911	895	906	927
**HP (mg/L)**		n.a.	25	25	10	25	25	25	25	25	25
**ICU**											
**Temperature (°C)**	45.7	44	41	44.5	47.2	44.5	35	46	44	43.7	47.5
**pH (pH units** **)**	7.4	7.2	7.7	7.7	7.5	7.7	7.7	7.8	7.7	7.7	7.7
**conductivity (µS/cm)**	1398	1380	790	882	869	885	923	904	892	907	923
**HP (mg/L)**		n.a.	25	25	10	25	25	25	25	25	25
**ONCO**											
**Temperature (°C)**	49.4	49	47.8	49.5	49.4	49.2	49	49.9	45.6	47.4	48.8
**pH (pH units** **)**	7.4	7.2	7.7	7.7	7.5	7.7	7.7	7.8	7.7	7.8	7.7
**conductivity (µS/cm)**	1399	1364	804	880	869	887	944	903	894	907	925
**HP (mg/L)**		n.a.	25	25	10	25	25	25	25	25	25
**OBST-GYN**											
**Temperature (°C)**	47.8	48	49.5	49.2	48.7	49.7	49.5	46	48	46.5	49.4
**pH (pH units)**	7.3	7.2	7.7	7.6	7.5	7.7	7.7	7.8	7.6	7.6	7.7
**conductivity (µS/cm)**	1398	1361	807	876	864	885	945	902	893	902	924
**HP (mg/L)**		n.a.	25	25	10	25	25	25	25	25	25
**RIC**											
**Temperature (°C)**	n.a.	n.a.	n.a.	n.a.	50.1	50.4	51	50.8	45.6	51.4	53.7
**pH (pH units** **)**	n.a.	n.a.	n.a.	n.a.	7.7	7.7	7.8	7.8	7.7	7.7	7.6
**conductivity (µS/cm)**	n.a.	n.a.	n.a.	n.a.	870	886	948	906	896	907	926
**HP (mg/L)**		n.a.	n.a.	n.a.	10	25	25	25	25	25	25
